# St. Olave's Union Infirmary

**Published:** 1895-09-21

**Authors:** 


### ST. OLAVE'S UNION INFIRMARY.

SIR,—As a ratepayer of Bermondsey, and one that takes a great interest in the welfare of our infirmary, I beg to thank you for bringing to public view the high-handed proceedings of our Guardians towards the matron, Miss Evans. After reading your remarks in THE HOSPITAL, I determined to interview one of my workmen who was an inmate in that institution for some time, but through the skill and care that he received has left and resumed his occupation. He informs me that since the new board came into office there has been an undercurrent at work to make the life of the matron perfectly wretched—taking the matron's authority out of her hands by an order that she shall have no voice in selecting her nurses. I see in the *South London Press* a very full account of the dispute between the Guardians and the nursing staff, stating that six nurses resigned on Thursday last, one of the Guardians remarking that it would not matter if 24 resigned. Do the Guardians recollect resolutions, viz., that they intended making the institution second to none for nursing, and that they intended training their own nurses? Sir, the surprise night visits are still in my memory, and I am surprised to be informed that one of the Guardians in the committee is still paying his addresses to one of the charge nurses. Is this the undercurrent? At the recent examination I understand that eleven nurses out of fifteen passed their examinations—three with distinction. Surely the doctors and matron are to be commended for such results, and a discourteous letter such as that the Guardians sent to Miss Evans after the above results does not speak well for the gentlemen (?) on the Board of Guardians. A L.G.B. inquiry is absolutely necessary to find out the truth of reports that are an open secret in Bermondsey, and I hope, sir, that you, in your powerful organ, will advocate this.—I am, sir, yours obediently, A RATEPAYER.

